# Ampere–Oersted field splitting of the nonlinear spin-torque vortex oscillator dynamics

**DOI:** 10.1038/s41598-022-14574-3

**Published:** 2022-06-23

**Authors:** Flavio Abreu Araujo, Chloé Chopin, Simon de Wergifosse

**Affiliations:** Institute of Condensed Matter and Nanosciences, Université catholique de Louvain, Place Croix du Sud 1, 1348 Louvain-la-Neuve, Belgium

**Keywords:** Condensed-matter physics, Spintronics

## Abstract

We investigate the impact of the DC current-induced Ampère–Oersted field on the dynamics of a vortex based spin-torque nano-oscillator. In this study we compare micromagnetic simulations performed using mumax$^3$ and our analytical model based on the Thiele equation approach. The latter is improved by adding two important corrections to the Thiele equation approach. The first is related to the magneto-static contribution and depends on the aspect ratio of the magnetic dot. The second is a full analytical description of the Ampère–Oersted field contribution. The model describes quantitatively the simulation results in the resonant regime as well as the impact of the Ampère–Oersted field. Depending on the relative orientation between the vortex in-plane curling magnetisation (chirality) and the Ampère–Oersted field a strong splitting phenomenon appears in the fundamental properties (frequency and vortex core position) of the nano-oscillator. Thus, we show that the Ampère–Oersted field should not be neglected as it has a high impact on the spin-torque vortex oscillator dynamics.

## Introduction

Most of the studies on spin-torque vortex oscillators (STVOs) neglect the Ampère–Oersted field (AOF) induced by the injected current (DC and/or AC)^1–5^. Moreover, it has also been shown that the AOF has an impact on the magnetisation dynamics of nanopillars^6^ as well as on the spin wave dynamics of spin-torque oscillators^7^. As far as the STVOs are concerned, the present study clearly shows that this contribution is not negligible and most importantly, is not avoidable by any means as it originates from the injected current responsible for the oscillatory excitation. Depending on the orientation of the polariser, the contributions arising from spin-transfer torque (STT)^8^ are different. To generate sustained oscillations thanks to the injected current, a DC and/or AC current is needed for a polariser with a perpendicular component. Whereas, for a polariser with a planar component, an AC current is required as oscillations are driven by the field-like torque (FLT)^9^ contribution in that case. For the sake of clarity, we choose to work with a perpendicular polariser and a DC current only.

Two improvements to the commonly used Thiele equation approach^10^ (TEA) are considered here. The first is related to the magneto-static contribution to the vortex energy as described by Gaididei et al.^1^. As the energy is highly dependent on the magnetic dot aspect ratio $\xi = h/(2R)$ (with *h* the thickness of the free-layer and *R* the dot radius) a significant correction of this contribution when $\xi$ is different from zero has to be considered. The second contribution to the Thiele equation approach is an analytical description of the AOF to the potential energy that has been already considered by Khvalkovskiy et al.^11^ and Dussaux et al.^12^. The latter studies report this contribution for a limited displacement of the vortex core reduced position $s = ||\mathbf {X}||/R = \sqrt{X^2+Y^2}/R$ ($0< s < 0.1$). The version we propose in this manuscript is accurate for the whole range of validity of the vortex core position in a circular dot, i.e., $0< s < 0.8$. The analytical model for the AOF contribution has already been successfully used in a previous study^13^. For $s > 0.8$, the vortex core is expelled from the magnetic dot, or the vortex reaches a critical velocity that induces a polarity switch and the core damps back to the dot centre^14,15^. In some cases, when the vortex core is expelled, a dynamic C-like state may also be stabilised and gives rise to sustained oscillations as shown by Wittrock et al.^16^.

## Methods

**Figure 1:**
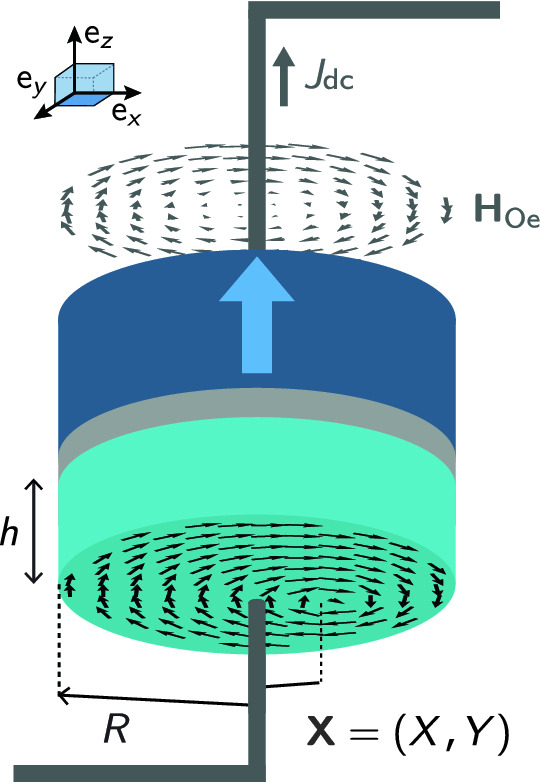
Illustration of the magnetic tunnel junction under study. The MTJ is a standard Py/MgO/SAF stack with a free permalloy (Py) layer dot, a nonmagnetic spacer (typically MgO), and a synthetic antiferromagnet (SAF) polariser that generates a perpendicular spin polarisation $p_{J}$. In this illustration, the Ampère–Oersted field $\mathbf {H}_{\mathrm{Oe}}$ is parallel to the dot in-plane magnetisation (chirality $C= +1$).

Micromagnetic simulations including the Ampère–Oersted field ($\mathbf{H} _{\mathrm{Oe}}$, see Fig. 1) using mumax$^3$ are performed^17^. The magnetic dot, i.e., the free layer of the magnetic tunnel junction (MTJ), under study has a radius *R* of 100 nm and a thickness *h* of 10 nm. The spacer is a tunnel barrier, typically MgO, and the polariser is a fixed polarised layer, with a unit vector polarisation $\mathbf {p}= (p_x, p_y, p_z)$, clamped by a synthetic antiferromagnet (SAF).

To perform the micromagnetic simulations, the magnetic dot is subdivided into 2.5 by 2.5 nm$^2$ squares in the *xy*-plane and two layers of 5 nm thickness each. This ensures that the micromagnetic cell dimensions are smaller than the characteristic exchange length $l_{\mathrm{ex}}= \sqrt{A/(2\pi M_{\mathrm{s}}^2)}$ for permalloy (about 5 nm). Without any external excitation, the magnetic ground state of such a dot is the so-called magnetic vortex. Typical material parameters for permalloy are used. For instance, the saturation magnetisation considered is $M_{\mathrm{s}}=800$ emu/cm$^3$ and the exchange stiffness constant is $A=1.07\cdot 10^{-6}$ erg/cm. The Gilbert damping constant is fixed at $\alpha _{\mathrm{G}} = 0.01$. The magneto-crystalline anisotropy is neglected. The DC current density $J_{\mathrm{dc}}$ injected in the MTJ under study ranges from 0 to 10 MA/cm$^2$, in the positive *z*-direction. The spin-current polarisation of the junction is chosen to be $p_J = 0.2$.

The vortex core position is initially slightly off-centred and is internally computed by mumax$^3$ while the frequency is extracted with a very accurate technique called “simple numerical instantaneous frequency approximation” (SNIFA)^18^ developed for that purpose. The current induced AOF is added to mumax$^3$ as an external magnetic field as there is no built-in implementation. The micromagnetic simulations are performed for a sufficient duration ( $> 1000$ to 3000 ns) to avoid to consider the transient regime. Furthermore, no bias external magnetic field is applied and the temperature is $T = 0$ K.

There are three different configurations studied in order to quantify the impact of the Ampère–Oersted field on the vortex dynamics for a vortex polarity $P = -1$:

1. without the Ampère–Oersted field ($\mathbf {H}_{\mathrm{Oe}}$) contribution,
2. with the $\mathbf {H}_{\mathrm{Oe}}$ parallel to the dot in-plane magnetisation (chirality $C = +1$),
3. with the $\mathbf {H}_{\mathrm{Oe}}$ anti-parallel to the dot in-plane magnetisation (chirality $C = -1$).

The first case will be referred to later on in the text with the label “$\mathrm{noOe}$”, the second with the label “$C^{+}$”, and the third with the label “$C^{-}$”.

In order to understand the physics of the STVO dynamics, the Thiele equation approach is used^10^. In this approach, the vortex core is considered as a quasi-particle and is represented by its in-plane position $\mathbf {X} = (X, Y)$ inside the magnetic dot. Thus, the STVO dynamics can be described by:

1 $$\begin{aligned} G(\mathbf {e}_z\times \dot{\mathbf {X}}) + D \dot{\mathbf {X}} =\frac{\partial W }{\partial \mathbf {X}} + \mathbf {F}^{\mathrm{ST}} \end{aligned}$$

where $G = -2 \pi P M_{\mathrm{s}}h / \gamma _{\mathrm{G}}$^19,20^, with $\gamma _{\mathrm{G}}=g|e|/(2m_e)\approx 1.76\cdot 10^{7}$ Oe$^{-1}$s$^{-1}$ the gyromagnetic ratio with *g* the electron spin *g*-factor, *e* the electron charge and $m_e$ the electron mass, $D = -\alpha _{\mathrm{G}}\eta |G|$ with $\eta = \frac{1}{2} \ln {(R/(2l_{\mathrm{ex}}))} + \frac{3}{8}$^11^, and $W = W^{\mathrm{ex}} + W^{\mathrm{Oe}} + W^{\mathrm{ms}}$ which are the three contributions of the energy. The restoring forces are expressed using their spring-like restoring force constants:

2 $$\begin{aligned} \frac{\partial (W^{\mathrm{ex}} + W^{\mathrm{Oe}} + W^{\mathrm{ms}}) }{\partial \mathbf {X}} =(k^{\mathrm{ex}}+ C J_{\mathrm{dc}}\kappa ^{\mathrm{Oe}}+ k^{\mathrm{ms}}) \mathbf {X} \end{aligned}$$

Three contributions are taken into account. The first one is related to the exchange energy and writes as follows^1,19^:

3 $$\begin{aligned} k^{\mathrm{ex}}(s) = (2 \pi )^2 h M_{\mathrm{s}}^2 \left( l_{\mathrm{ex}}/ R\right) ^2/(1-s^2) \end{aligned}$$

The second contribution is related to the Ampère–Oersted field and has been calculated under the two vortex ansatz (TVA). The following analytical expression is obtained (see the Supplementary material for details):

4 $$\begin{aligned} \kappa ^{\mathrm{Oe}}(s) = \frac{8 \pi ^2}{75} M_{\mathrm{s}}R h \left( 1 - \frac{4}{7} s^2 - \frac{1}{7} s^4 - \frac{16}{231} s^6 - \frac{125}{3003} s^8 \right) \end{aligned}$$

Finally, to enhance the precision of the model, a third contribution is considered. Indeed, a correction to the contribution of the magneto-static energy $W^{\mathrm{ms}}$ is made. The latter has been calculated by Gaididei et al.^1^ under the two vortex ansatz and gives $W^{\mathrm{ms}}(\xi , s) = 4 M_{\mathrm{s}}^2 h^2 R s^2 \cdot \Theta (\xi , s)$ which is the magneto-static energy for a given aspect ratio $\xi$ and reduced vortex position *s*. There exists an analytical solution^1^ for $\Theta (\xi , s)$ but only when $\xi = 0$ and $s = 0$.

In this study, $\xi = 0.05$, thus, a different method is used instead (see Supplementary material). Let us note that this method is applicable to any $\xi$, including $\xi = 0$. First, a Monte Carlo (MC) integration technique (non-deterministic) is used to obtain values of $W^{\mathrm{ms}}(\xi , s) \equiv W^{\mathrm{ms}}_\xi (s)$ for a given $\xi$ and for *s* ranging between 0 and 1. Then $W^{\mathrm{ms}}_\xi (s)$ is fitted with a power law and the resulting expression is derived. As a result, the contribution of the magneto-static energy for a given aspect ratio $\xi$ of the magnetic dot can be modelled as:

5 $$\begin{aligned} k_{\xi }^{\mathrm{ms}}(s) = \frac{8 M_{\mathrm{s}}^2 h^2}{R} \Lambda _{0,\xi }\left( 1 + a_\xi s^2 + b_\xi s^4 + c_\xi s^6 \right) \end{aligned}$$

The coefficients $\Lambda _{0,\xi }, a_\xi , b_\xi$ and $c_\xi$ given for $\xi = 0$ and $\xi = 0.05$ are reported in the Supplementary material. For $\xi = 0$, $\Lambda _{0,\xi =0}= 1.7424$ which is in good agreement with the analytical results by Guslienko et al.^20^ with $8 \Lambda _{0,\xi =0}\approx 40\pi /9$ (diff. less than 0.2%). To simplify, $k^{\mathrm{ms}}_\xi$ is replaced by $k^{\mathrm{ms}}$ in the rest of the manuscript.

The spin-transfer torque component is composed of three terms: $\mathbf {F}^{\mathrm{ST}}=\mathbf {F}^{\mathrm{ST}}_{\perp }+\mathbf {F}^{\mathrm{ST}}_{\parallel } +\mathbf {F}^{\mathrm{ST}}_{\mathrm{FLT}}$. The first two terms are respectively the perpendicular and parallel contribution of the Slonczewski spin-torque^8^ while the third one is the Zhang & Li field-like-torque (FLT)^9^.

In this study, a perpendicular polariser, i.e., $\mathbf{p} = (0, 0, 1)$, is used, as already investigated by Ivanov and Zaspel^21^ within TEA. So, $\mathbf {F}^{\mathrm{ST}}_{\parallel }$ ($= 0$, proportional to $\mathbf{p} _{x,y} = (0, 0)$ in this case^12^) and $\mathbf {F}^{\mathrm{ST}}_{\mathrm{FLT}}$ ($=\kappa ^{\mathrm{ST}}_{\mathrm{FLT}}J_{\mathrm{dc}}(\mathbf{e} _z\times \mathbf{p} ) = 0$) are equal to zero and only the $\mathbf {F}^{\mathrm{ST}}_\perp$ contributes to the dynamics. $\mathbf {F}^{\mathrm{ST}}_\perp = \kappa ^{\mathrm{ST}}_{\perp }J_{\mathrm{dc}}(\mathbf {e}_z \times \mathbf {X})$, where $\kappa ^{\mathrm{ST}}_{\perp }= \pi a_J M_{\mathrm{s}}h p_z$ with $a_J = p_J \hslash / (2|e|M_{\mathrm{s}}^{\mathrm{ref}}h)$,^11^, $p_z = 1$ and $M_{\mathrm{s}}^{\mathrm{ref}}$ = 800 emu/cm$^3$.

From Eq.(1) one can rewrite the STVO dynamics as:

6 $$\begin{aligned} \left[ {\begin{array}{cc} D &{} -G \\ G &{} D \\ \end{array} } \right] \left[ {\begin{array}{c} \dot{X} \\ \dot{Y} \\ \end{array} } \right] =\left[ {\begin{array}{cc} k &{} -\kappa ^{\mathrm{ST}}_{\perp }J_{\mathrm{dc}}\\ \kappa ^{\mathrm{ST}}_{\perp }J_{\mathrm{dc}}&{} k\\ \end{array} } \right] \left[ {\begin{array}{c} X \\ Y \\ \end{array} } \right] \end{aligned}$$

where $k = k^{\mathrm{ex}}+ C \kappa ^{\mathrm{Oe}}J_{\mathrm{dc}}+ k^{\mathrm{ms}}$. This equation can be reduced to a homogeneous system of linear first-order differential equations:

7 $$\begin{aligned} \left[ {\begin{array}{c} \dot{X} \\ \dot{Y} \\ \end{array} } \right] =\underbrace{\left[ {\begin{array}{cc} \Gamma &{} -\omega \\ \omega &{} \Gamma \\ \end{array} } \right] }_{\bar{\bar{\Omega }}} \left[ {\begin{array}{c} X \\ Y \\ \end{array} } \right] \end{aligned}$$

where the parameters $\Gamma$ and $\omega$ are given as follows:

8 $$\begin{aligned} \Gamma&= \frac{D\left( k^{\mathrm{ex}}+ C J_{\mathrm{dc}}\kappa ^{\mathrm{Oe}}+ k^{\mathrm{ms}}\right) +G J_{\mathrm{dc}}\kappa ^{\mathrm{ST}}_{\perp }}{D^2+G^2} \end{aligned}$$

9 $$\begin{aligned} \omega&= \frac{D J_{\mathrm{dc}}\kappa ^{\mathrm{ST}}_{\perp }- G \left( k^{\mathrm{ex}}+ C J_{\mathrm{dc}}\kappa ^{\mathrm{Oe}}+ k^{\mathrm{ms}}\right) }{D^2+G^2} \end{aligned}$$

This shows that the dynamics of our STVO is described by the simple harmonic oscillator equation: $\dot{\mathbf {X}} = \bar{\bar{\Omega }}\mathbf {X}$. Considering $\Gamma \rightarrow 0$ and $\omega$ as being constant one can prove that the solution of this system writes:

10 $$\begin{aligned} \left[ {\begin{array}{c} X(t) \\ Y(t) \\ \end{array} } \right] =||\mathbf {X}||e^{\Gamma t} \left[ {\begin{array}{c} \sin {(\omega t)} \\ -\cos {(\omega t)} \\ \end{array} } \right] \end{aligned}$$

In general $\Gamma$ and $\omega$ are not constant, but in the steady-state oscillating regime they effectively are. Important properties of the STVO can be derived from this regime of oscillation.

From the first parameter, *i.e.*, $\omega$, it is straightforward to identify the vortex gyrotropic frequency of oscillation as $f^{\mathrm{STVO}}(J_{\mathrm{dc}})=\omega (J_{\mathrm{dc}})/(2\pi )$. The second parameter, *i.e.*, $\Gamma$, is responsible for the transient regime. When $\Gamma <0$, the transient dynamics is in the damping regime leading to $s=0$ giving rise to the resonant regime. When $\Gamma > 0$, the transient dynamics is in the driving regime ($s > 0$) and leads to the steady-state oscillating regime where $\Gamma = 0$ and *s* becomes constant. The first critical current $J_{\mathrm{c}1}$ can thus be analytically defined by the limit when $\Gamma = 0$ and $s = 0$. Mathematically this condition translates to:

11 $$\begin{aligned} \Gamma (s=0) = \frac{D\left( k^{\mathrm{ex}}_0+CJ_{\mathrm{c}1}\kappa ^{\mathrm{Oe}}_0+k^{\mathrm{ms}}_0\right) +GJ_{\mathrm{c}1}\kappa ^{\mathrm{ST}}_{\perp }}{D^2+G^2}=0 \end{aligned}$$

The critical current is thus given by the following expression:

12 $$\begin{aligned} J_{\mathrm{c}1}= \frac{-D(k^{\mathrm{ex}}_0 + k^{\mathrm{ms}}_0)}{D C \kappa ^{\mathrm{Oe}}_0+ G \kappa ^{\mathrm{ST}}_{\perp }} \end{aligned}$$

The corrections allow having a model valid for *s* up to 0.8 which is the limit of the vortex existence. As mentioned already, for $s>0.8$ the vortex becomes unstable and switches polarity in this case, *i.e.*, for $\xi = 0.05$. The vortex core position *s* depends on the current density $J_{\mathrm{dc}}$. Thus, one can define a second critical current $J_{\mathrm{c}2}$ which represents the current density above which the vortex core polarity switches. Once the vortex switches polarity, the model well describes the dynamics of the STVO providing the opposite polarity (damping regime). Thus, the critical current $J_{\mathrm{c}2}$ establishes the limit between the steady-state oscillating regime and the second resonant regime due to the reversal of the core polarity.

To summarise, there are two resonant regimes ($s = 0$) and one steady-state regime (in-plane gyrotropic oscillation of the vortex core with $s > 0$). To be in the steady-state oscillating regime, two conditions need to be fulfilled: $J_{\mathrm{dc}}P p_z<0$ and $\pm |J_{\mathrm{c}1}| \lessgtr \pm |J_{\mathrm{dc}}| \lessgtr \pm |J_{\mathrm{c}2}|$. For both resonant regimes, at least one of these conditions is not satisfied and the existence of the second critical current $J_{\mathrm{c}2}$ originates from the fact that when $\pm |J_{\mathrm{dc}}| \gtrless \pm |J_{\mathrm{c}2}|$ then $s > 0.8$ and the vortex core switches polarity so that the first condition is not fulfilled anymore. Corrections to the other terms (*s*-dependence of *G* and *D* for instance) are being considered under a data-driven approach and will be discussed in a future communication.

## Discussion

**Figure 2:**
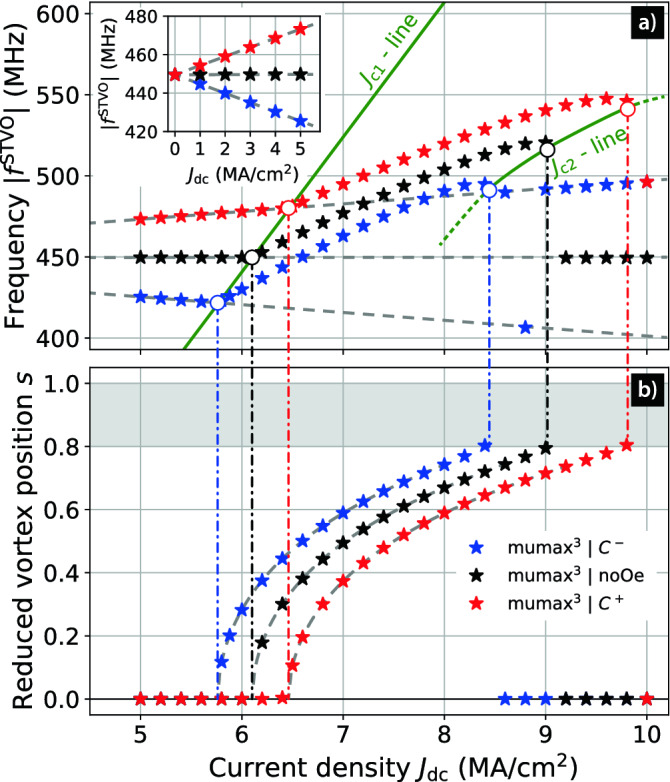
Vortex dynamical properties vs. DC current excitation. (**a**) The absolute values of the vortex gyrotropic frequency $f^{\mathrm{STVO}}$ and (**b**) the vortex reduced position *s* as a function of the DC current density $J_{\mathrm{dc}}$. The colours black, red and blue correspond to simulations without AOF ($\mathrm{noOe}$), with AOF and $C = +1$ ($C^{+}$) and with AOF and $C = -1$ ($C^{-}$), respectively. The frequency is fitted with a linear function of $J_{\mathrm{dc}}$ in the first resonant regime ($J_{\mathrm{dc}}< J_{\mathrm{c}1}$) for each configuration. The resulting fits are plotted with grey dashed lines. The critical current $J_{\mathrm{c}1}$ is extracted from $s(J_{\mathrm{dc}})$ and gives rise to the first green $J_{\mathrm{c}1}$-line. The $J_{\mathrm{c}1}$-line represents the transition between the first resonant regime and the steady-state oscillating regime. The $J_{\mathrm{c}1}$-line links the $f^{\mathrm{STVO}}(J_{\mathrm{c}1})$ points originating from the micromagnetic data and is also well approximated by the analytical counterpart $f^{\mathrm{STVO}} = J_{\mathrm{dc}}\cdot \kappa ^{\mathrm{ST}}_{\perp }/ (2\pi D)$. The $J_{\mathrm{c}1}$ and $J_{\mathrm{c}2}$ values are plotted across both sub-figures by corresponding dash-dotted lines. The $J_{\mathrm{c}2}$-line represents the transition from the steady-state oscillating regime to the second resonant regime as for $J_{\mathrm{dc}}>J_{\mathrm{c}2}$, the vortex core polarity switches from $P=-1$ to $P=+1$ and $J_{\mathrm{dc}}P p_z$ becomes positive.

The critical current and the vortex gyrotropic frequency given by the analytical model and micromagnetic simulations are compared. The critical current is extracted from the micromagnetic simulations by the following method. First, the reduced vortex core position *s* is plotted as a function of the current density (see Fig. 2b). Then, the points for $0< s < 0.8$ (*i.e.*, when the steady-state is reached and before reaching the limits of vortex stability) are fitted after an interpolation which increases the fit precision. The function used for the fit is $s(J_{\mathrm{dc}}) = (a_0 + a_1 J_{\mathrm{dc}}+ a_2 J_{\mathrm{dc}}^2) \sqrt{(J_{\mathrm{dc}}/ J_{\mathrm{c}1}- 1)}$ (see Fig. 2b). The critical current $J_{\mathrm{c}1}$ is the value of the fit when $s = 0$ and is strongly impacted by the AOF. Indeed, $J_{\mathrm{c}1}$ depends on the chirality as previously shown by Dussaux et al.^12^ and one can see that $J_{\mathrm{c}1}^{C^{-}}<J_{\mathrm{c}1}^{\mathrm{noOe}} < J_{\mathrm{c}1}^{C^{+}}$. The critical current $J_{\mathrm{c}1}$, for both chiralities, is very close to the values determined by the TEA as shown in Table 1. The ratio between the critical currents $J_{\mathrm{c}1}$ and $J_{\mathrm{c}2}$ is about 1.5 as also reported by Guslienko et al.^22^ and detailed in Table 1.

**Table 1:** Comparison of $J_{\mathrm{c}1}$ and $J_{\mathrm{c}2}$ given by Eq. (12) under TEA and micromagnetic simulations (MMS) after fitting the *J*-dependence of *s* (see Fig. 2b) for the different relative orientations of the AOF and the in-plane magnetisation.

	$C^+$	noOe	$C^-$
$J_{\mathrm{c}1}^{\mathrm{TEA}}$ (MA/cm$^2$)	6.88	6.45	6.08
$J_{\mathrm{c}1}^{\mathrm{MMS}}$ (MA/cm$^2$)	6.47	6.11	5.77
$J_{\mathrm{c}2}^{\mathrm{MMS}}$ (MA/cm$^2$)	9.83	9.10	8.43
$J_{\mathrm{c}2}^{\mathrm{MMS}}/J_{\mathrm{c}1}^{\mathrm{MMS}}$	1.52	1.50	1.46

**Figure 3:**
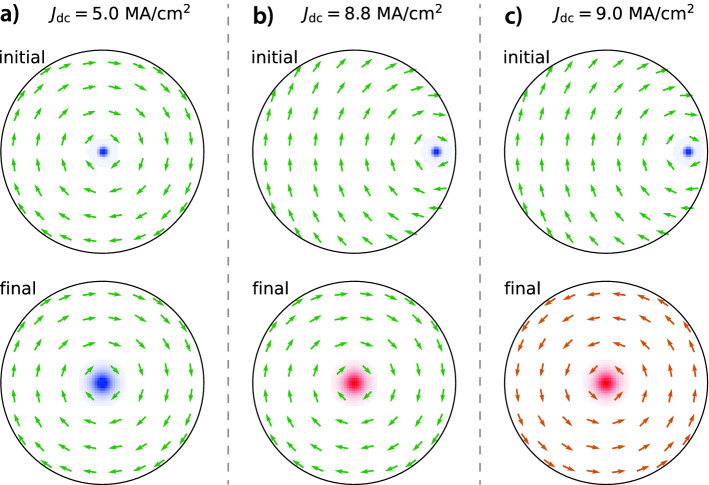
Magnetisation of the magnetic dot at the beginning (initial state) and at the end (final state) of the micromagnetic simulations. The *x*-*y* component of the magnetisation is represented by green (clockwise chirality) or orange (counterclockwise chirality) arrows while the *z* component is represented by a colour code, i.e., dark blue for $m_z=-1$, white for $m_z=0$, and dark red for $m_z=+1$. The vortex core is initially placed at $x=1$ nm and $y=0$ nm for $J_{\mathrm{dc}}< J_{\mathrm{c}1}$ and at $x=80$ nm and $y=0$ nm for $J_{\mathrm{dc}}> J_{\mathrm{c}2}$. The initial state of the vortex is $C=-1$ and $P=-1$. To simplify the visualisation, the arrows represent the averaged magnetisation over multiple cells. (**a**) For $C^{-}$ and $J_{\mathrm{dc}}=$ 5.0 MA/cm$^2$, the vortex final state is damped back to the dot centre as at least one of the conditions for steady-state oscillations is not fulfilled, namely $J_{\mathrm{dc}}< J_{\mathrm{c}1}$. Here, the chirality and the polarity do not change. (**b**) For $C^{-}$ and $J_{\mathrm{dc}}>J_{\mathrm{c}2}$ with $J_{\mathrm{dc}}=$ 8.8 MA/cm$^2$, only the vortex core polarity switches from $P=-1$ (blue) to $P=+1$ (red). Here, the same chirality as in the beginning nucleates. (**c**) For $C^{-}$ and $J_{\mathrm{dc}}=$ 9.0 MA/cm$^2$, both the vortex core polarity switches from $P=-1$ (blue) to $P=+1$ (red) and the vortex chirality switches as it is initially clockwise ($C=-1$) and becomes counterclockwise ($C=+1$) at the end of the simulation.

The splitting due to the AOF can be clearly seen in Fig. 2 as it has a strong impact on *s*, $J_{\mathrm{c}1}$, $J_{\mathrm{c}2}$, and $|f^{\mathrm{STVO}}|$ depending on the vortex chirality. In addition, one can see that to the right of the $J_{\mathrm{c}2}$-line, *i.e.*, for $s > 0.8$, the vortex polarity switches and the chirality of the vortex initially at $C^-$ also switches for all points except for $J_{\mathrm{dc}}=$ 8.8 MA/cm$^2$ (see Fig. 2a and Fig. 3). Indeed, the AOF favours the chirality leading to an in-plane magnetisation parallel to $\mathbf {H}_{\mathrm{Oe}}$, i.e., the chirality $C^+$ in our case.

To simplify the expression of $\omega$ given by Eq. (9) , one can consider $D \ll G$ and the resonant regime ($J_{\mathrm{dc}}< J_{\mathrm{c}1}$) leading to $s = 0$. Thus, $\omega$ can be reduced to $\omega = -(k^{\mathrm{ex}}+ C J_{\mathrm{dc}}\kappa ^{\mathrm{Oe}}+ k^{\mathrm{ms}}) / G$. In this regime $k^{\mathrm{ex}}$, $\kappa ^{\mathrm{Oe}}$, $k^{\mathrm{ms}}$ and *G* can be considered as constant and *G* can be rewritten as $G = -P|G|$ as all components of *G* are positive excepted *P* which can be either $+1$ or $-1$. The angular frequency $\omega$ can then be approximated by the following linear equation: $\omega = P(2\pi \alpha _JJ_{\mathrm{dc}}+ \omega _0)$ with $2\pi \alpha _J= C \kappa ^{\mathrm{Oe}}_0/ |G|$ and $\omega _0= (k^{\mathrm{ex}}_0 + k^{\mathrm{ms}}_0) / |G|$. As $\omega _0$ is positive because $k^{\mathrm{ex}}_0 > 0$ and $k^{\mathrm{ms}}_0 > 0$, for $\omega _0\gg |2\pi \alpha _JJ_{\mathrm{dc}}|$ the sign of $\omega$ is given by the sign of $P\omega _0$ and thus by *P*. In other words, the polarity *P* determines the vortex rotational direction.

From Fig. 2a, $f_0 = \omega _0/(2\pi )$ and $\alpha _J$ are extracted with a linear fit and the results are given in Table 2. The frequency $f_0 = \omega _0/ (2\pi ) = (k^{\mathrm{ex}}_0 + k^{\mathrm{ms}}_0) / (2 \pi |G|)$ is almost the same for the three curves as anticipated because it does not depend on $\kappa ^{\mathrm{Oe}}_0$. In addition, the sign of the slope $\alpha _J= C\kappa ^{\mathrm{Oe}}_0/(2\pi |G|)$ depends of the chirality ($C = \pm 1$) and is close to zero without AOF^23^ as expected from the analytical model.

These results show the importance of the AOF which must be taken into account to precisely describe the dynamics of STVO in the resonant regime as well as in the steady-state oscillating regime (here by applying a DC current). In addition, the impact of the AOF on the STVO dynamics depends on the magnetic dot properties including both material and geometry.

**Table 2:** Comparison of $f_0$ and $\alpha _J$ between the TEA and the fit from micromagnetic simulations (MMS) ($|f| = f_0 + \alpha _JJ_{\mathrm{dc}}$).

	$\mathrm{noOe}$		$C^{+}$		$C^{-}$	
	TEA	MMS	TEA	MMS	TEA	MMS
$f_0$ (MHz)	492.53	449.51	492.53	449.57	492.53	449.56
$\alpha _J$ (MHz cm$^2$/A)	0	0.03	4.69	4.74	− 4.69	− 4.82

**Figure 4:**
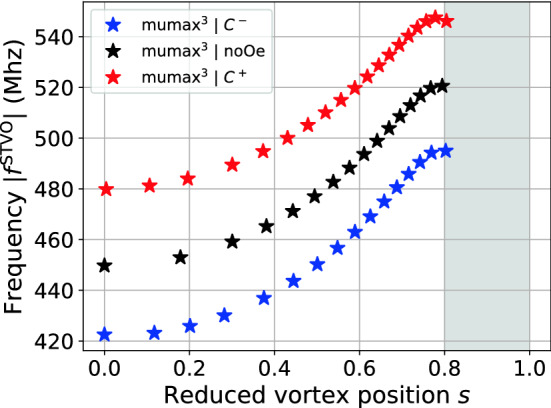
STVO intrinsic property. The gyrotropic frequency $|f^{\mathrm{STVO}}|$ as a function of the reduced vortex core position *s*. Only data in the steady-state oscillating regime is shown here.

The injected DC current responsible for the steady-state oscillations and thus $s > 0$ should be seen as a way to modify the vortex position *s*. The intrinsic property of the dynamics of the STVO is rather given by the gyrotropic frequency vs. the reduced vortex core position *s* as shown in Fig. 4, considering only the data from the steady-state oscillating regime. Indeed, the gyrotropic frequency depends only on *s* for each configuration as it modifies the energy *W* of the dot. Here *s* depends on $J_{\mathrm{dc}}$ but there are others means for modifying the vortex position *s* (in-plane field, AC current, stray field antenna, *etc*.). The splitting is due to the fact that the energy *W* changes when the chirality changes as shown in Eq. (2) while keeping the DC current orientation constant with the adequate sign for obtaining the steady-state regime. As illustrated in Fig. 1, the curling AOF is counterclockwise when $J_{\mathrm{dc}}> 0$.

Figure 4, shows the relation between the reduced vortex core position *s* and the frequency in the steady-state oscillating regime. The latter increases with *s*, as seen in the study by Dussaux et al^12^. Depending on the applied current, one can tune the frequency on a range of roughly 75 MHz, for each case. A maximum appears just before the vortex polarity reversal which happens at $s\approx 0.8$. A clear splitting due to the AOF field is exhibited. Its magnitude, evaluated with $|f_{C^+}-f_{C^-}|$, decreases slightly for increasing *s*. Our model predicts this behaviour as $k^{\mathrm{Oe}}(s) = C J_{\mathrm{dc}}(s) \cdot \kappa ^{\mathrm{Oe}}(s)$, the spring-like constant associated to the AOF, decreases constantly with *s* (see Fig. 2b).

As mentioned previously, the resonant regime is quantitatively described by the improved analytical model. However, the steady-state oscillating regime, as shown in Fig. 4, is at this stage only described qualitatively (not shown here) and further work on the *s*-dependence of the *G* and *D* terms is needed. The important message of Fig. 4 is that for a given energy configuration of such a STVO where the chirality $C = \pm 1$ is a key parameter, micromagnetic simulations show that the frequency is intrinsically depending on the vortex core position. The same holds for the resonant regime.

## Conclusion

In conclusion, a splitting is observed due to the AOF which depends on the chirality of the vortex. The improved analytical model which is valid for $0 \le s \le 0.8$, thanks to corrections of the magneto-static and AOF contributions, is coherent with the micromagnetic simulation results for $J_{\mathrm{c}1}$ and $\omega$ in the resonant regime. This shows the importance of the AOF for understanding the complex nonlinear dynamics of vortex based nano-oscillators. The splitting shown and modelled here represents a supplementary degree of freedom of such oscillators with potentially a high impact on future applications. For instance, we believe that our analytical model is a first step towards making a fast model for the simulation of STVOs in the framework of reservoir computing^24^ and build complex hardware-based neuromorphic computing devices.

## Supplementary Information

Supplementary Information.

## Data Availability

The datasets generated during and/or analysed during the current study are available from the corresponding author on reasonable request.
