# Supplementary material for: Ampere–Oersted field splitting of the nonlinear spin-torque vortex oscillator dynamics
Source: Sci Rep. 2022 Jun 23;12:10605. doi: 10.1038/s41598-022-14574-3 (PMC9226115; doi:10.1038/s41598-022-14574-3)
Supplement: Supplementary file 1 — Supplementary Information. [file 41598_2022_14574_MOESM1_ESM.pdf]

Supplementary material:

Ampere-Oersted field splitting of the nonlinear spin-torque vortex oscillator dynamics

Flavio ABREU ARAUJO[1,*], Chloé CHOPIN[1], and Simon de WERGIFOSSE[1]

[1]Institute of Condensed Matter and Nanosciences, Université catholique de Louvain, Place Croix du Sud 1, 1348 Louvain-la-Neuve, Belgium
[*]flavio.abreuaraujo@uclouvain.be

The harmonic oscillator equation

Our oscillator dynamics is described by the following homogeneous system of linear first-order differential equations:

$$\dot{\mathbf{X}} = \bar{\bar{\Omega}}\mathbf{X} \tag{1}$$

where the matrix $\bar{\bar{\Omega}}$ is given as:

$$\bar{\bar{\Omega}} = \begin{bmatrix} \Gamma & -\omega \\ \omega & \Gamma \end{bmatrix} \tag{2}$$

Considering Γ and ω as being constant one can derive time-dependent solutions for the vortex core position $(X(t), Y(t))$. This assumption is verified in a steady-state regime of oscillation. The eigenvalues λ of the system are calculated from its characteristic equation:

$$\lambda^2 - 2\Gamma\lambda + (\Gamma^2 + \omega^2) = 0 \tag{3}$$

The latter has been obtained from $\det(\bar{\bar{\Omega}} - \lambda\bar{\bar{\mathbf{I}}}) = 0$. The two solutions of Eq. (3) are $\lambda = \Gamma \pm i\omega$. Using Euler's formula one can prove that the coordinates of the vortex core are given as:

$$X(t) = e^{\Gamma t}[(C_1 + C_2)\cos(\omega t) + i(C_1 - C_2)\sin(\omega t)]$$
$$Y(t) = e^{\Gamma t}[-i(C_1 - C_2)\cos(\omega t) + (C_1 + C_2)\sin(\omega t)]$$

If the two constants are fixed such as $C_1 = -C_2 = -\frac{i}{2}||\mathbf{X}||$ one finally obtains:

$$\begin{bmatrix} X(t) \\ Y(t) \end{bmatrix} = ||\mathbf{X}||e^{\Gamma t}\begin{bmatrix} \sin(\omega t) \\ -\cos(\omega t) \end{bmatrix} \tag{4}$$

Ampère-Oersted field contribution to the Thiele equation

Ampère's law (SI):

$$\begin{aligned} \oint_c \mathbf{B} \cdot d\ell &= 4\pi k_{\mathrm{m}} I_c \\ &= \mu_0 I_c \end{aligned}$$

where k_{m} is the magnetic force constant ($\mu_0/(4\pi)$) and μ_0 is the magnetic constant [$4\pi \cdot 10^{-7}$ T/(A/m)].

Let's take the case of an infinite wire of radius R with a uniform current I flowing through it. I_c represents the current flowing inside the path c of integration.

Inside the wire ($r \leqslant R$): $I_c = I\pi r^2/(\pi R^2) = Ir^2/R^2 = \pi J r^2$, where $J = I/(\pi R^2)$ is the current density. Outside the wire ($r > R$): $I_c = I$.

$$\Rightarrow \oint_c \mathbf{B} \cdot d\ell = B 2\pi r = \mu_0 I_c$$

For $r \leqslant R$ (inside the wire): $B 2\pi r = \mu_0 \pi |J| r^2$ and $B(r) = \frac{\mu_0 |J|}{2} r$

For $r > R$ (outside the wire): $B 2\pi r = \mu_0 \pi |J| R^2$ and $B(r) = \frac{\mu_0 |J|}{2} R^2 \frac{1}{r}$

The Oersted field vector obtained is: $\mathbf{B}(\mathbf{r}) = B(r)\left(\cos\left(\theta + \sigma\frac{\pi}{2}\right), \sin\left(\theta + \sigma\frac{\pi}{2}\right), 0\right) = B(r)\mathbf{b}(\mathbf{r})$, where $\mathbf{r} = r(\cos\theta, \sin\theta, 0)$ or $\mathbf{r} = (r, \theta)$, $\sigma = \text{sign}(J) = \pm 1$ and $\mathbf{b}(\mathbf{r})$ is the Oersted field unit vector. Inside a magnetic dot of radius R and thickness h, the potential energy due to the current induced Oersted field and a shifted magnetic vortex state of magnetisation distribution $\mathbf{M}(\mathbf{r}, \mathbf{X})$ with $\mathbf{X} = (\rho, \varphi)$ being the vortex core position is given by:

$$W^{\text{Oe}} = -\int_V \mathbf{B}(\mathbf{r}) \cdot \mathbf{M}(\mathbf{r}, \mathbf{X}) dV,$$

where V corresponds to the magnetic dot volume.

In SI units $B_{\text{SI}}(r) = \dfrac{\mu_0 |J|}{2} r = Q_{\text{SI}} |J| r$ [T], with $Q_{\text{SI}} = \dfrac{\mu_0}{2}$ [T·m/A], and $J \Rightarrow$ [A/m^2].

In CGS units $B_{\text{CGS}}(r) = \dfrac{\pi |J|}{5} r = Q_{\text{CGS}} |J| r$ [G], with $Q_{\text{CGS}} = \dfrac{\pi}{5}$ [G·cm/A], and $J \Rightarrow$ [A/cm^2].

$\mathbf{B}(\mathbf{r}) = B(r)\mathbf{b}(\mathbf{r}) = Q|J| r \mathbf{b}(\mathbf{r})$ and $\mathbf{M}(\mathbf{r}, \mathbf{X}) = M_{\text{s}} \mathbf{m}(\mathbf{r}, \mathbf{X})$, where M_{s} is the spontaneous magnetisation and $\mathbf{m}(\mathbf{r}, \mathbf{X})$ is the normalised magnetisation profile (distribution) of the magnetic vortex. The potential energy W^{Oe} yields:

$$W^{\text{Oe}} = -Q|J| M_{\text{s}} \int_V r \mathbf{b}(\mathbf{r}) \cdot \mathbf{m}(\mathbf{r}, \mathbf{X}) dV.$$

As $\mathbf{b}(\mathbf{r}) = (\cos\phi_1, \sin\phi_1, 0)$, with $\phi_1 = \theta + \sigma\pi/2$, has no out-of-plane component, the z-component (vortex core shape) of $\mathbf{m}(\mathbf{r}, \mathbf{X})$ is not contributing, so $\Rightarrow \mathbf{m} = (\cos\phi_2, \sin\phi_2, 0)$, *i.e.*,$\Rightarrow \mathbf{m}(\mathbf{r}, \mathbf{X}) = (\cos[\phi_2(\mathbf{r}, \mathbf{X})], \sin[\phi_2(\mathbf{r}, \mathbf{X})], 0))$.

The in-plane profile $\phi_2(\mathbf{r}, \mathbf{X})$ of an off-centred vortex is well described within the "Two Vortex Ansatz" TVA (or "Image Vortex Ansatz", IVA) as no side charges are created (unlike the "Single Vortex Ansatz" also called the "Rigid Vortex Ansatz"):

$$\phi_2^{\text{TVA}}(\mathbf{r}, \mathbf{X}) = \arg(\mathbf{r} - \mathbf{X}) + \arg(\mathbf{r} - \mathbf{X}_{\text{I}}) - \varphi + C\pi/2$$

where $\mathbf{X}_{\text{I}} = \left(R^2/\rho, \varphi\right)$ are the image vortex coordinates ($\|\mathbf{X}_{\text{I}}\| = R^2/\rho^2 \|\mathbf{X}\|$) and C is the vortex chirality ($= \pm 1$).

One obtains explicitly:

$$\phi_2^{\text{TVA}}(\mathbf{r}, \mathbf{X}) = \tan^{-1}\left(\frac{r\sin\theta - \rho\sin\varphi}{r\cos\theta - \rho\cos\varphi}\right) + \tan^{-1}\left(\frac{\rho r\sin\theta - R^2\sin\varphi}{\rho r\cos\theta - R^2\cos\varphi}\right) - \varphi + C\frac{\pi}{2}$$

The cylindrical symmetry of the energy evaluation of an off-centred vortex with respect to the Oersted field makes it independent from φ, so we choose to take $\varphi = 0$:

$$\phi_2^{\text{TVA}}(r, \rho, \theta) = \tan^{-1}\left(\frac{r\sin\theta}{r\cos\theta - \rho}\right) + \tan^{-1}\left(\frac{\rho r\sin\theta}{\rho r\cos\theta - R^2}\right) + C\frac{\pi}{2}$$

Finally, using reduced variables $\eta = r/R$ and $s = \rho/R$ we obtain:

$$\phi_2^{\text{TVA}}(\eta, s, \theta) = \tan^{-1}\left(\frac{\eta\sin\theta}{\eta\cos\theta - s}\right) + \tan^{-1}\left(\frac{s\eta\sin\theta}{s\eta\cos\theta - 1}\right) + C\frac{\pi}{2}$$

$$\begin{aligned} W^{\text{Oe}} &= -Q|J| M_{\text{s}} \int_V r\mathbf{b}(\mathbf{r}) \cdot \mathbf{m}(\mathbf{r}, \mathbf{X}) dV \\ &= -Q|J| M_{\text{s}} \int_0^h \int_0^{2\pi} \int_0^R r^2 \mathbf{b}(\mathbf{r}) \cdot \mathbf{m}(\mathbf{r}, \mathbf{X}) dr d\theta dz \\ &= -Q|J| M_{\text{s}} h \int_0^{2\pi} \int_0^R r^2 \mathbf{b}(\mathbf{r}) \cdot \mathbf{m}(\mathbf{r}, \mathbf{X}) dr d\theta \end{aligned}$$

The previous transformation is due to the fact that there is no z-dependence.

As $\eta = r/R$, $dr = Rd\eta$:

$$\begin{aligned} W^{\text{Oe}} &= -Q|J|M_s h \int_0^{2\pi}\int_0^1 R^2 \frac{r^2}{R^2}\mathbf{b}(\mathbf{r})\cdot\mathbf{m}(\mathbf{r},\mathbf{X}) R d\eta d\theta \\ &= -Q|J|M_s h R^3 \int_0^{2\pi}\int_0^1 \eta^2 \mathbf{b}(\mathbf{r})\cdot\mathbf{m}(\mathbf{r},\mathbf{X}) d\eta d\theta \end{aligned}$$

$$\begin{aligned} \mathbf{b}(\mathbf{r})\cdot\mathbf{m}(\mathbf{r},\mathbf{X}) &= (\cos\phi_1, \sin\phi_1, 0)\cdot(\cos\phi_2^{\text{TVA}}, \sin\phi_2^{\text{TVA}}, 0) \\ &= \cos\phi_1\cos\phi_2^{\text{TVA}} + \sin\phi_1\sin\phi_2^{\text{TVA}} \\ &= \cos\left(\phi_1 - \phi_2^{\text{TVA}}\right) \end{aligned}$$

$$\begin{aligned} \phi_1 - \phi_2^{\text{TVA}} &= \theta + \sigma\frac{\pi}{2} - \tan^{-1}\left(\frac{\eta\sin\theta}{\eta\cos\theta - s}\right) - \tan^{-1}\left(\frac{s\eta\sin\theta}{s\eta\cos\theta - 1}\right) - C\frac{\pi}{2} \\ &= \theta + (\sigma - C)\frac{\pi}{2} - \tan^{-1}\left(\frac{\eta\sin\theta}{\eta\cos\theta - s}\right) - \tan^{-1}\left(\frac{s\eta\sin\theta}{s\eta\cos\theta - 1}\right) \end{aligned}$$

Using $\theta = \tan^{-1}\left(\frac{\sin\theta}{\cos\theta}\right)$ and $\tan^{-1}(a) \pm \tan^{-1}(b) = \tan^{-1}\left(\frac{a \pm b}{1 \mp ab}\right)$:

$$\begin{aligned} \phi_1 - \phi_2^{\text{TVA}} &= (\sigma - C)\frac{\pi}{2} + \tan^{-1}\underbrace{\left(\frac{\sin\theta}{\cos\theta}\right)}_{\lambda} - \tan^{-1}\underbrace{\left(\frac{s\eta^2\sin 2\theta - \eta(1+s^2)\sin\theta}{s\eta^2\cos 2\theta - \eta(1+s^2)\cos\theta + s}\right)}_{\mu} \\ &= (\sigma - C)\frac{\pi}{2} + \tan^{-1}\left(\frac{\lambda - \mu}{1 + \lambda\mu}\right) \end{aligned}$$

$$\begin{aligned} \mathbf{b}(\mathbf{r})\cdot\mathbf{m}(\mathbf{r},\mathbf{X}) &= \cos\left[\underbrace{(\sigma - C)\frac{\pi}{2}}_{A} + \underbrace{\tan^{-1}\left(\frac{\lambda - \mu}{1 + \lambda\mu}\right)}_{B}\right] \\ &= \cos A\cos B - \underbrace{\sin A\sin B}_{=\,0\ (A\,=\,0 \text{ or } \pi)} \\ &= \cos\left((\sigma - C)\frac{\pi}{2}\right)\cos\left(\tan^{-1}\left(\frac{\lambda - \mu}{1 + \lambda\mu}\right)\right) \\ &= \sigma C \frac{1}{\sqrt{1 + \left(\frac{\lambda - \mu}{1 + \lambda\mu}\right)^2}} \end{aligned}$$

$$\mathbf{b}(\mathbf{r})\cdot\mathbf{m}(\mathbf{r},\mathbf{X}) = \sigma C \frac{\eta\left(s^2+1\right) - s\cos(\theta)\left(\eta^2+1\right)}{\sqrt{\left(\eta\left(s^2+1\right) - s\cos(\theta)\left(\eta^2+1\right)\right)^2 + s^2\sin(\theta)^2\left(\eta^2-1\right)^2}}$$

Finally, the potential energy writes ($\sigma|J| = J$):

$$W^{\text{Oe}}(s) = -QJCM_s h R^3 \int_0^{2\pi}\int_0^1 \Theta(s,\eta,\theta) d\eta d\theta, \tag{5}$$

where
$$\Theta(s,\eta,\theta) = \frac{\eta^3\left(s^2+1\right)-\eta^2 s\cos\left(\theta\right)\left(\eta^2+1\right)}{\sqrt{\left(\eta\left(s^2+1\right)-s\cos\left(\theta\right)\left(\eta^2+1\right)\right)^2+s^2\sin^2\left(\theta\right)\left(\eta^2-1\right)^2}}.$$

There are two possibilities to evaluate Eq. (5) to obtain the $s=\rho/R$ dependence of W^{Oe}. The first is to numerically integrate it and then to fit the result to a power law in s. The second possibility is to compute the Taylor expansion (TE) of the integrand of Eq. (5) and then solve it analytically. Both techniques are considered and compared hereafter. Figure S1 shows the numerical resolution of the integral in Eq. (5) in blue. The 6th order power law fit and the 10th order Taylor expansion are represented in blue and red dashed lines, respectively.

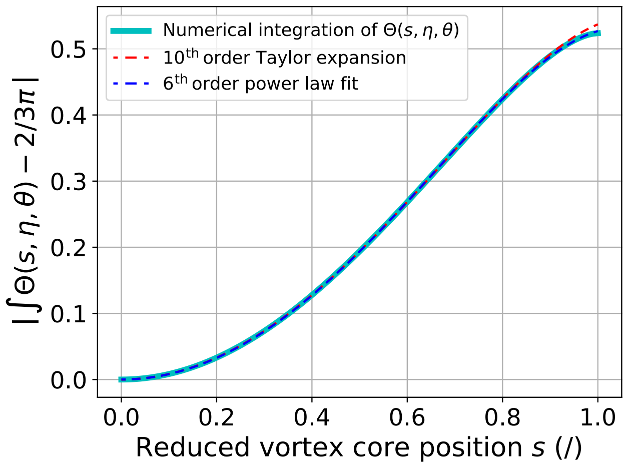

Figure S1. Evolution of the double integral in Eq. (5) vs. reduced vortex core position s. The thick light blue line corresponds to the numerical integration, the dashed blue line is the 6th order fit over the numerical data and the dashed red line is the result after analytical integration of the 10th order Taylor expansion.

It should be noticed that $2/3\pi$ has been subtracted from the overall value of the integral shown in Fig. S1 in such a way to start at zero and avoid the evaluation of this parameter during the fit. The fitting coefficients are given as follows:

$$W^{\mathrm{Oe}}_{\mathrm{fit}}(s) = -QJCM_{\mathrm{s}}hR^3\left(\frac{2}{3}\pi - 0.827s^2 + 0.180s^4 + 0.119s^6\right). \tag{6}$$

The 10th order Taylor expansion gives:

$$W^{\mathrm{Oe}}_{\mathrm{TE}}(s) = -QJCM_{\mathrm{s}}hR^3\left(\frac{2}{3}\pi - \frac{4\pi}{15}s^2 + \frac{8\pi}{105}s^4 + \frac{4\pi}{315}s^6 + \frac{16\pi}{3465}s^8 + \frac{20\pi}{9009}s^{10}\right). \tag{7}$$

Figure S2 shows the error level between the numerical integration data and both, the fit [Eq. (6)] and the 10th order Taylor expansion [Eq. (7)].

An important consequence of the error level comparison is the fact that the 10th order TE is much more accurate than the fit for $s \leqslant 0.78$, but for $s > 0.78$ the fit maintains a lower error level.

As mentioned in the main text of this manuscript, the vortex is unstable for $s > 0.8$, so the Taylor expansion is a better choice for modelling the Ampère-Oersted field contribution to the Thiele equation.

The current induced Oersted field acts as a restoring force and one can thus consider:

$$W^{\mathrm{Oe}}(X) = \frac{1}{2}k^{\mathrm{Oe}}X^2, \tag{8}$$

where X $(=\rho)$ is the vortex core orbital radius and k^{Oe} is the restoring force constant (also called vortex stiffness parameter). The corresponding restoring force magnitude writes:

$$F^{\mathrm{Oe}}(X) = -\frac{\partial W^{\mathrm{Oe}}(X)}{\partial X} = -k^{\mathrm{Oe}}(X)X. \tag{9}$$

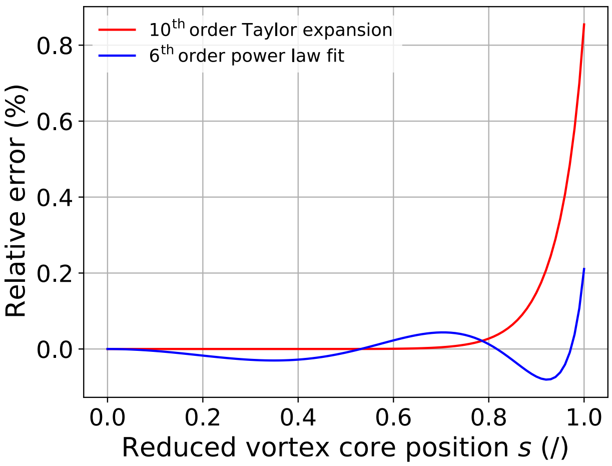

Figure S2. Evolution of the relative error level between the numerical integration data and both, the fit [6th order power law, Eq. (6)] and the 10th order Taylor expansion [Eq. (7)].

The energy W^{Oe} in Eqs. (6) and (7) is already depending on s, so we can rewrite Eq. (9) as follows to consider the force in terms of the reduced vortex core position s:

$$\begin{aligned}\frac{F^{\mathrm{Oe}}(s)}{R} &= -\frac{1}{R}\frac{\partial W^{\mathrm{Oe}}(s)}{\partial s}\frac{\partial s}{\partial X} = -k^{\mathrm{Oe}}(s)\frac{X}{R},\\ &= -\frac{1}{R^2}\frac{\partial W^{\mathrm{Oe}}(s)}{\partial s} = -k^{\mathrm{Oe}}(s)s.\end{aligned}$$

Eqs. (6) and (7) then give the following results for the respective restoring force constants:

$$\begin{aligned}k^{\mathrm{Oe}}_{\mathrm{fit}}(s) &= QJCM_s hR\left(1.654 - 0.720s^2 - 0.714s^4\right)\\ &= QJCM_s hR\cdot 1.654\left(1 - 0.435s^2 - 0.432s^4\right),\end{aligned}$$

and

$$\begin{aligned}k^{\mathrm{Oe}}_{\mathrm{TE}}(s) &= QJCM_s hR\left(\frac{8\pi}{15} - \frac{32\pi}{105}s^2 - \frac{24\pi}{315}s^4 - \frac{128\pi}{3465}s^6 - \frac{200\pi}{9009}s^8\right)\\ &= QJCM_s hR\frac{8\pi}{15}\left(1 - \frac{4}{7}s^2 - \frac{1}{7}s^4 - \frac{16}{231}s^6 - \frac{125}{3003}s^8\right).\end{aligned}$$

In addition, one can redefine the spring-like restoring force constant associated to the Ampère-Oersted field as $k^{\mathrm{Oe}}(s) = \kappa^{\mathrm{Oe}}(s)JC$ for the sake of clarity. This transformation, used in the main text of this manuscript, allows to highlight the impact of the current density and the chirality on the oscillator dynamics.

Finally, the oscillation frequency contribution of the Oersted field is given by $\omega^{\mathrm{Oe}} = k^{\mathrm{Oe}}/G$.

$$\omega^{\mathrm{Oe}}_{\mathrm{fit}}(s) = -\frac{1.654\cdot QJCR\gamma_{\mathrm{G}}}{2\pi P}\left(1 - 0.435s^2 - 0.432s^4\right),$$

and

$$\omega^{\mathrm{Oe}}_{\mathrm{TE}}(s) = -\frac{4QJCR\gamma_{\mathrm{G}}}{15P}\left(1 - \frac{4}{7}s^2 - \frac{1}{7}s^4 - \frac{16}{231}s^6 - \frac{125}{3003}s^8\right).$$

Magneto-static contribution to the Thiele equation

The magneto-static contribution $W^{\rm ms}(\xi,s)$ to the Thiele equation has been calculated by Gaididei et *al.*[1] under the "Two Vortex Ansatz" and gives the following equation:

$$W^{\rm ms}(\xi,s) = 4M_s^2 h^2 R s^2 \cdot \Theta(\xi,s), \tag{10}$$

with

$$\Theta(\xi,s) = \int_0^1 d\zeta \int_0^1 d\eta \int_0^1 d\eta' \int_0^{2\pi} d\varphi \int_0^{2\pi} d\varphi' \Xi(\zeta,\eta,\eta',\varphi,\varphi',\xi,s),$$

$$\Xi(\zeta,\eta,\eta',\varphi,\varphi',\xi,s) = \frac{\eta\eta'(1-\zeta)\Psi(\eta,s,\varphi)\Psi(\eta',s,\varphi+\varphi')}{\sqrt{\eta^2+\eta'^2-2\eta\eta'\cos\varphi'+4\xi^2\zeta^2}},$$

and

$$\Psi(\eta,s,\varphi) = \frac{\eta\sin\varphi}{\sqrt{s^2+\eta^2-2s\eta\cos\varphi}\sqrt{1+s^2\eta^2-2s\eta\cos\varphi}},$$

where

$$s = \frac{X}{R}, \xi = \frac{h}{2R}, \eta = \frac{r}{R}, \eta' = \frac{r'}{R}.$$

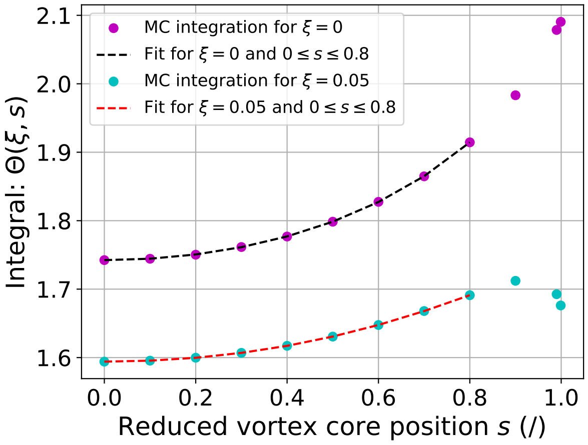

Figure S3. Evolution of the integral $\Theta(\xi,s)$ (see Eq. (10)) vs. the reduced vortex position s. The magenta dots represent the numerical Monte Carlo integration for $\xi = 0$ whereas the cyan dots represent the Monte Carlo integration for $\xi = 0.05$. The dashed lines correspond to a 6th order power law fit of the numerical data in the range of stability of the vortex, *i.e.*,$0 \le s \le 0.8$. The black and red dashed lines correspond to $\xi = 0$ and $\xi = 0.05$, respectively. The coefficients after derivation of the power law model are given in Table S1.

There is no analytical solution for Eq. (10) and a deterministic numerical integration is not feasible. So, we performed a Monte Carlo (MC) integration (non-deterministic) with guaranteed absolute precision of 10^{-4}.[1] It should be noticed that an analytical solution exists when $\xi = 0$ and $s = 0$. Gaididei *et al.*[1] obtained $\Theta(0,0) = 2\pi(2\mathscr{C}-1)/3 \backsimeq 1.742393$ where $\mathscr{C} = 0.5\int_0^1 K(x)dx \backsimeq 0.916$ with $K(x)$ the elliptic integral of the first kind.

The MC integration for $\Theta(\xi = 0, s)$ gives after fitting the numerical data and deriving the corresponding power law:

$$k^{\rm ms}_{\xi=0.0}(s) = \frac{8M_s^2h^2}{R} 1.7424\left(1+0.254s^2-0.026s^4+0.320s^6\right). \tag{11}$$

Even if the practical magnetic dot geometries give rise to very small ξ values, the latter are not zero. For instance in this manuscript, $R = 100$ nm and $h = 10$ nm, so $\xi = h/(2R) = 0.05$. The Monte Carlo integration for $\Theta(\xi = 0.05, s)$ gives rise to:

$$k^{\rm ms}_{\xi=0.05}(s) = \frac{8M_s^2h^2}{R} 1.5941\left(1+0.175s^2+0.065s^4-0.054s^6\right). \tag{12}$$

[1]The library used for the MC integration is GAIL version 2.0 (Guaranteed Automatic Integration Library) developed by the Illinois Institute of Technology.

The mean relative overestimation of Eq. (11) compared to Eq. (12) is about 10.3%. It goes from 9.3% at $s = 0$ to 13.2% at $s = 0.8$. As the oscillation frequency contribution of the magneto-static energy is given by $\omega^{\mathrm{ms}} = k^{\mathrm{ms}}/G$, the vortex core gyrotropic frequencies computed using $\xi = 0$ instead of $\xi = 0.05$ in this case give rise to a mean error of about 10.3%.

ξ	$\Lambda_{0,\xi}$	a^{ξ}	b^{ξ}	c^{ξ}
0.0	1.7424	0.254	−0.026	0.320
0.05	1.5941	0.175	0.065	−0.054

Table S1. Values of the coefficients of the k^{ms} term after the fit and derivation according to the power law model given in the main text.

References

1. Gaididei, Y., Kravchuk, V. P. & Sheka, D. D. Magnetic vortex dynamics induced by an electrical current. Int. J. Quantum Chem. **110**, 83–97 (2010).
